# Hearing Loss and Kidney Dysfunction: Finding a Unifying Diagnosis

**DOI:** 10.1155/2013/858963

**Published:** 2013-02-21

**Authors:** Praveena Iruku, Puja Karanth, Hannah Tiu, Charity Kankam, Khaldoon Shaheen

**Affiliations:** ^1^Department of Medicine, Case Western Reserve University, St. Vincent Charity Medical Center, Cleveland, OH 44115, USA; ^2^Department of Hospital Medicine, Institute of Medicine, Cleveland Clinic, Cleveland, OH 44195, USA; ^3^Department of Nephrology, Case Western Reserve University, St. Vincent Charity Medical Center, Cleveland, OH 44115, USA; ^4^Cleveland Clinic Lerner College of Medicine of Case Western Reserve University, Department of Hospital Medicine, Institute of Medicine, Cleveland Clinic, 9500 Euclid Avenue, A13, Cleveland, OH 44195, USA

## Abstract

Microscopic polyangiitis (MPA) is a systemic vasculitis that affects small caliber vessels, with renal and lung compromise. Diagnosis can be challenging; timely diagnosis and treatment are important to prevent devastating complication, particularly renal failure. We present a case of a patient with microscopic polyangiitis presented with renal and pulmonary involvements with concomitant sensorineural hearing loss. We provide diagnostic, therapeutic, and prognostic keys to microscopic polyangiitis.

## 1. Introduction

Microscopic polyangiitis (MPA) is a systemic vasculitis that affects small caliber vessels, with renal and lung compromise. Diagnosis can be challenging; timely diagnosis and treatment are important to prevent devastating complication, particularly renal failure. We present a case of a patient with microscopic polyangiitis presented with renal and pulmonary involvements with concomitant sensorineural hearing loss. We provide diagnostic, therapeutic, and prognostic keys to microscopic polyangiitis.

## 2. The Case

**Figure 1:**
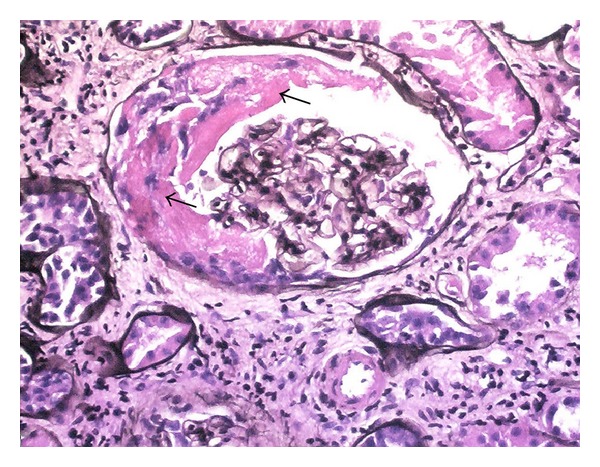
Histopathology slide showing signs of MPA in kidney biopsy. Fibrinous crescent filling approximately half of the glomerulus (arrows) with interstitial chronic inflammation. Jones stain/methenamine silver stain, 200x.

**Figure 2:**
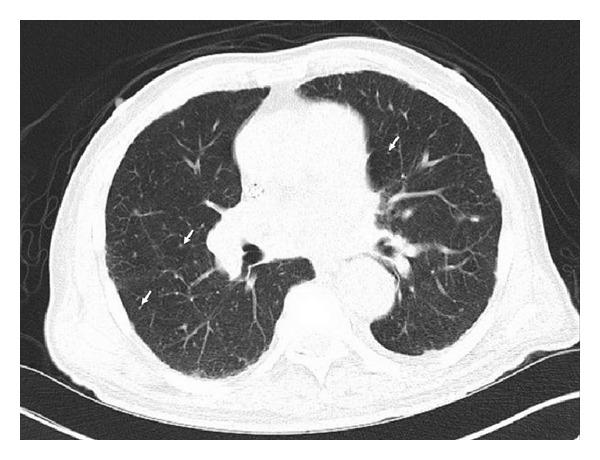
CT scan revealed the diffuse of tiny bilateral pulmonary nodules (arrows) secondary to granulomatous disease particularly with large calcified granulomas with mediastinal and hilar lymphadenopathy. There was evidence of pulmonary arterial hypertension.

This is a 79-year-old African American man presented to our inpatient service with three month history of progression of renal dysfunction associated with generalized weakness, loss of appetite, and weight loss of about 20 pounds. He denied any chest pain, cough, shortness of breath, abdominal pain, night sweats, fever, bleeding per rectum, hematuria, or dysuria. Review of systems was significant for hearing deficit on the right side for the last 2 months. His medical history was significant for hypertension and dyslipidemia. He was taking lisinopril and simvastatin. His occupation was fixing water pumps for about 20 years. His social history was significant for smoking (10 pack years) and occasional alcohol intake. He denied use of illicit drugs. On examination, temperature was 36.2°C, blood pressure was 117/64, respiratory rate was 14/minute, heart rate was 73 beats/min, and BMI was 22.8. Other findings were significant for pallor and bilateral sensorineural hearing loss; right side is more than the left. A mildly enlarged nontender prostate was found on rectal exam. Otherwise, rest of the systemic examination was unremarkable. Stool for fecal occult blood was negative. Laboratory examination revealed a leukocyte count of 6000 cells/mm^3^, hemoglobin of 9.3 with a hematocrit of 26.7 (normochromic normocytic anemia), and platelets of 267,000. ESR was 70 mm/hr (reference 0–20 mm/hr), and CRP was 45 mg/L (reference range 0.0–3.0 mg/L). Basic metabolic panel revealed a potassium of 5.4 mmol/L (3.5–5.0), sodium of 134 mmol/L (136–142), chloride of 99 (98–107), bicarbonate of 20 (23–32), BUN of 91 (7–18), and creatinine of 9.3 (0.5–1.5); a marked increment of serum creatinine levels from 0.65 mg/dL during the last 3 months had been found. Urinalysis disclosed nephritic urinary sediments (RBCs 51–75/high-power field (HPF); leukocytes, 10/15/HPF; and granular casts, 2-3/HPF   with proteinuria 4 g/day. PSA was 0.83, and TSH was 2.907 (0.5–5.0). Ultrasound of kidneys and bladder showed normal size kidneys with no hydronephrosis, calculi, or masses. Further testing included that complement levels were normal. Serum and urine protein electrophoresis were negative. Syphilis serology, antihepatitis B/C antibodies, HIV panel, antiglomerular basement membrane antibody, and proteinase-3 ANCA were negative. Serum antineutrophil cytoplasmic autoantibody, a perinuclear pattern (p-ANCA) was elevated at a titer of 1 : 640, and myeloperoxidase antibodies (MPO) were 1 : 100. Kidney biopsy was done which revealed presence of crescents with sclerosing glomerulonephritis (Figure 1). Our patient was considered to have pauci-immune focal sclerosing glomerulopathy with crescentic features and a positive p-ANCA suggesting microscopic polyangiitis (MPA). In rolling out any concomitant pulmonary involvement, a CT scan of the lungs was done and showed bilateral pulmonary nodules (Figure 2) which can be seen in patients with microscopic polyangiitis. For induction of remission, he was commenced on intravenous methylprednisone at a dose of 10 mg/kg and cyclophosphamide monthly intravenous therapy for six months. After five doses of methylprednisone intravenously, an oral course of steroids was started. The patient did well after completing six doses of intravenous cyclophosphamide, both clinically and immunologically his energy levels returned to normal. His ANCA titer reverted to normal. His sensorineural hearing loss showed marked improvement. As his serum creatinine decreased to 0.9 mg/dL and remained stable, he was continued on maintenance immunosuppressive therapy with no evidence of relapse.

## 3. Discussion

Microscopic polyangiitis (MPA) is a rare systemic vasculitis that involves many vital organs. It is generally seen in older people but can be seen in any age group [1]. It is very difficult to distinguish from Wegener's granulomatosis (granulomatosis with polyangiitis) based on clinical grounds. The most common clinical manifestations include glomerulonephritis, weight loss, mononeuritis multiplex, fevers, and variety of cutaneous findings [2, 3]. Other clinical manifestations include oral or palatal ulcers, sinusitis, purulent rhinorrhea, hemoptysis, hematuria, leukocytoclastic vasculitis, sesorineural hearing loss, pulmonary fibrosis, and pulmonary hypertension [2, 3]. In contrast alveolar hemorrhage is seen in a minority of patients. Diagnosis is mainly established by clinical manifestations, antibodies, and biopsy. P-ANCA antibodies are positive in approximately 90% of patients [4]. Myeloperoxidase antibodies are primarily associated with MPA, whereas PR3 antibodies are primarily seen in Wegener's granulomatosis (WG) [5]. The organ with active involvement is usually biopsied. Biopsy of the kidneys typically shows focal segmental glomerulonephritis with crescents that is pauci-immune on immunoflorescence examination [6]. It is differentiated from WG by the absence of granulomas, but the absence of granulomas on transbronchial specimens is not adequate to exclude diagnosis of WG [7].

It is extremely important to diagnose and initiate therapy in patients with diagnosis of MPA. The rates of remission are high. The management includes induction therapy which is typically done with cyclophosphamide and methylprednisone and induction of remission usually takes 2 to 6 months [8]. Maintenance therapy is done with less toxic drugs, which include methotrexate or azathioprine for about 12 to 18 months [8].

The impairment of hearing followed the clinical and laboratory manifestations of systemic vasculitis (i.e., the constitutional manifestations, inflammatory signs, and urinary abnormalities) and other causes of hearing loss, such as otitis media or mastoiditis, were not found in our patient. MRI brain was done also and was negative. Furthermore, the corticosteroid and immunosuppressive therapies effectively reduced the hearing loss as well as the activities of these manifestations of vasculitis. Therefore, the sensorineural hearing loss in this case was thought to be caused by autoimmune inner ear disorder associated with MPA [9]. Hair cells in the inner ear are thought to be sensitive to ischemic changes; thus, reversal of the circulatory disturbances due to systemic vasculitis usually results in improvement in the sensorineural hearing loss. Moreover, sensorineural hearing loss associated with MPA has been described in very few patients [10]. This type of sensorineural hearing loss can also present in other systemic vasculitides. It has been reported in the patients with Bechet's disease, Cogan's syndrome, giant cell arteritis, mixed cryoglobulinemia, polyarteritis nodosa, Takayasu's arteritis, and Wegener's granulomatosis (WG) [11]. However, in WG, conductive hearing loss is the most common audiologic finding. It is caused by otitis media, mastoiditis or, less frequently, by direct WG involvement of the middle ear. Sensorineural hearing loss is less common. The cause is unclear; suggested mechanisms include cochlear nerve compression by adjacent granuloma, cochlear immune-complex deposition, and local vasculitis that involve cochlear vessels [12, 13]. Cogan's syndrome is a rare disorder of an unknown origin characterized by inflammatory eye disease and vestibulo-auditory symptoms, which primarily affects young white adults manifests as nonsyphilitic interstitial keratitis (IK), acute onset sensorineural hearing loss and vestibular symptoms such as Ménière's disease, and progressive hearing loss up to deafness within 2 years [14]. As the patients with MPA are commonly elderly like our patient, the hearing loss related to this vasculitis may often have been overlooked and misdiagnosed it as presbyacusis.

## 4. Conclusion

Our case highlights the complexity of diagnosing microscopic polyangiitis (MPA) which is a rare systemic vasculitis that involves many vital organs. Sensorineural hearing loss associated with MPA, caused by autoimmune inner ear disorder, has been described in very few patients in the literature. Early diagnosis and treatment of MPA is crucial. Furthermore, reversal of the circulatory disturbances due to systemic vasculitis in MPA usually results in improvement in the concomitant sensorineural hearing loss.
